# Development and Validation of a Multidimensional Predictive Model for 28-Day Mortality in Patients with Post-Traumatic Acute Respiratory Distress Syndrome

**DOI:** 10.3390/jcm15052073

**Published:** 2026-03-09

**Authors:** Piao Zhang, Chengcheng Sun, Renchao Zou, Li Zhou, Chunling Jiang

**Affiliations:** 1 Department of Anaesthesiology, West China Hospital, Sichuan University, Chengdu 610041, China; alien-piao@zju.edu.cn (P.Z.); zlmz@wchscu.cn (L.Z.); 2 Department of Emergency Medicine, Chengdu Third People’s Hospital, Chengdu 610000, China; sunchengcheng@comen.com; 3 Department of Hepatobiliary Surgery, The Second Affiliated Hospital of Kunming Medical University, Kunming 650101, China; zourenchao@kmmu.edu.cn

**Keywords:** trauma, acute respiratory distress syndrome, nomogram, predictive model

## Abstract

**Objective:** To develop and validate a multidimensional nomogram for predicting 28-day all-cause mortality in patients with post-traumatic acute respiratory distress syndrome (ARDS). **Methods:** A retrospective analysis was conducted on 667 post-traumatic ARDS patients from the MIMIC-IV database, divided into training (n = 466) and validation (n = 201) cohorts (7:3). LASSO regression combined with the Boruta algorithm was used to screen variables and construct a nomogram. Model performance was evaluated by AUROC, calibration curves, and decision curve analysis (DCA) with SHAP analysis to identify core predictors. **Results:** Ten variables (e.g., lactate, platelet transfusion units, D-dimer) were selected and used to construct the nomogram model. The nomogram showed superior discriminative ability (AUROC = 0.848 in training set, 0.846 in validation set) compared with SOFA, APACHE II scores, and machine learning models (XGBoost, random forest). Calibration curves confirmed good agreement between predicted and actual risks, and DCA indicated better clinical net benefit. SHAP analysis identified lactate and platelet transfusion units as core risk factors and albumin and base excess trauma as protective factors. **Conclusions:** The nomogram has excellent predictive efficacy and interpretability, providing a reliable tool for clinical intervention in post-traumatic ARDS patients.

## 1. Introduction

Trauma is a common cause of death in severely injured patients, secondary to hemorrhagic shock and cardiopulmonary dysfunction [1,2]. Approximately 10–20% of trauma patients may develop severe respiratory complications [3]. Among them, acute respiratory distress syndrome (ARDS) is correlated to substantial morbidity and mortality in intensive care unit (ICU) [4]. Post-trauma ARDS is triggered by multiple factors, including direct thoracic trauma and/or indirect injury and treatment strategies such as massive transfusion, fluid overload, and ventilator-induced acute lung injury [2,4,5]. Systemic inflammatory response syndrome, destruction of the alveolar–capillary barrier, and multiple organ dysfunction result in a faster progression and poorer prognosis in patients with post-traumatic ARDS, and presenting a significant challenge for clinical treatment. The aetiologies of post-trauma ARDS are heterogeneous, and early diagnosis and treatment may influence the outcome. Therefore, the prompt and precise recognition of high-risk cases with ARDS following trauma along with the execution of stratified intervention measures would tremendously reduce mortality and enhance prognosis.

Although the early diagnosis of post-traumatic-ARDS is crucial for improving clinical outcomes, specific diagnostic tools remain lacking in clinical practice. Currently, the commonly used clinical assessment tools such as the Sequential Organ Failure Assessment (SOFA) and Acute Physiology and Chronic Health Evaluation II (APACHE II) can reflect the overall severity of ARDS, but they have obvious limitations [6,7]. These methods do not focus on the unique pathophysiological features of post-traumatic ARDS (such as the initiation of traumatic inflammation, the interaction between coagulation and inflammation, etc.), which results in their specificity being insufficient for predicting the prognosis of trauma subgroups [8]. These methods rely on multiple dimensional static indicators, making it difficult to capture the dynamic changes in the patient’s condition after trauma [9]. Moreover, it fails to integrate key information such as imaging and biomarkers, thus limiting its clinical practicability and accuracy. In recent years, although certain machine learning models have demonstrated great advantages in predicting the prognosis of ARDS, they are often difficult to apply in the complex scenarios of trauma due to their “black-box” nature, which lacks clinical interpretability [10]. The nomogram, as a visual multi-factor prediction tool, combines prediction accuracy with clinical interpretability [11] and has been verified in various studies on the prognosis of critical illnesses [11,12]. However, there are still significant research gaps in the nomogram prediction model for the “post-traumatic ARDS” subgroup, which is highly heterogeneous. Currently, relevant studies are mostly limited to single-center, small-sample designs. Trauma-specific indicators (such as trauma severity scores, radiological lung injury severity, and coagulation function indicators) have not been fully integrated into variable selection. Furthermore, there is a lack of systematic performance comparisons with traditional scoring systems and machine learning models, nor has SHapley Additive exPlanations (SHAP) analysis been used to elucidate the mechanism of trauma-related factors. These shortcomings result in insufficient clinical credibility and practicality in the established models. Therefore, this study aims to develop an intuitive and user-friendly nomogram prediction tool based on clinical parameters within the first 24 h of admission for trauma patients. Utilizing the MIMIC-IV database, this study enrolled adult ICU patients with traumatic injuries and concurrent ARDS. We identified key predictors of 28-day all-cause mortality using LASSO regression combined with the Boruta algorithm. Then, a multidimensional nomogram with high discriminative power and strong interpretability was developed. Subsequently, we validated the model’s performance in training and validation cohorts via receiver operating characteristic (ROC) curves, calibration curves, and decision curve analysis (DCA), with simultaneous comparative analyses against traditional scoring systems (e.g., SOFA, APACHE II) and machine learning models (e.g., random forest, XGBoost). Finally, SHAP analysis was employed to elucidate the contribution mechanism of trauma-related factors to mortality risk. This study aims to provide a reliable predictive tool for the early identification of high-risk patients with post-traumatic ARDS and the formulation of individualized intervention strategies, thereby ultimately improving patient prognosis.

## 2. Materials and Methods

### 2.1. Data Source

The data were extracted from the Medical Information Mart for Intensive Care IV (MIMIC-IV) version 3.0 database. This openly accessible repository contains comprehensive medical information from the ICU of the Massachusetts Institute of Technology Beth Israel Deaconess Medical Center, covering patient stays between 2008 and 2022 [13]. The researchers completed the corresponding CITI training and obtained the data usage license (Certificate No: 72709155).

### 2.2. Study Population

**Figure 1:**
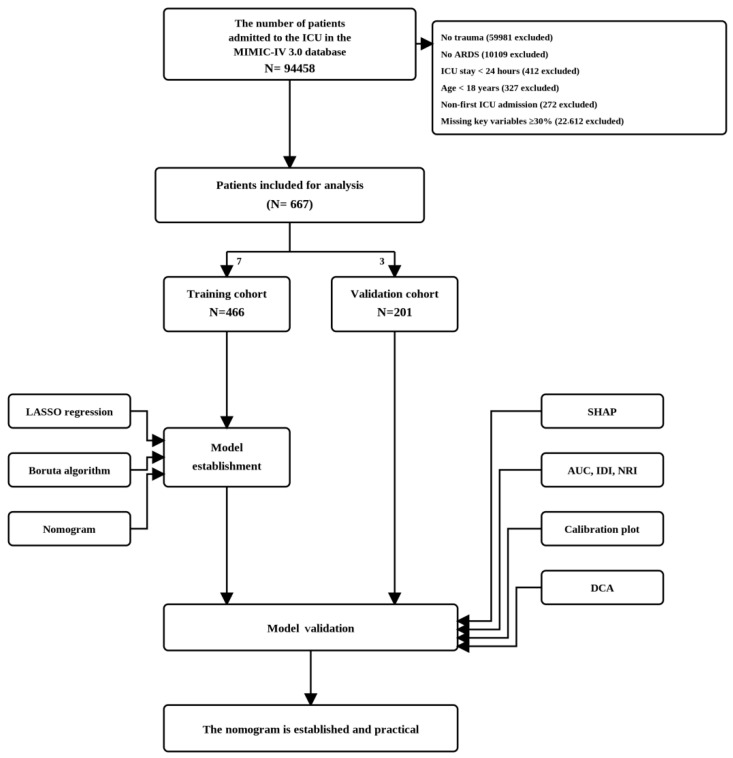
Overall study flowchart.

The inclusion criteria of this study are as follows: (1) Age ≥ 18 years; (2) First admission to the ICU; (3) Trauma-induced ARDS that fulfills the diagnostic criteria of the Berlin Definition during ICU stay [14]. The exclusion criteria include: (1) Patients admitted for non-traumatic causes; (2) Trauma patients without ARDS; (3) ICU length of stay < 24 h; (4) Missing proportion of key variables ≥ 30%; (5) Duplicate ICU admission records. After screening according to the aforementioned criteria, a total of 667 patients with post-traumatic ARDS were finally enrolled with independent and complete data for each case (Figure 1).

### 2.3. Variables Extraction

Multi-dimensional characteristics of patients were extracted using SQL [15]. Of these, a total of 69 variables are predictor variables used for modeling, and 1 is the outcome variable (mortality_28d), which indicates whether death occurred within 28 days. These variables cover multiple domains as detailed below: (1) Demographic and admission information: subject_id, hadm_id, stay_id, age, gender, ethnicity, admission type, admission source, and insurance type; (2) Day-1 vital signs: heart rate (HR), systolic blood pressure (SBP), diastolic blood pressure (DBP), mean arterial pressure (MAP), respiratory rate (RR), body temperature (T), pulse oxygen saturation (SpO_2_), and fraction of inspired oxygen (FiO_2_); (3) Laboratory tests: hemoglobin, platelet, white blood cell count (WBC), albumin, blood urea nitrogen (BUN), creatinine (Cr), electrolytes [sodium (Na^+^), potassium (K^+^)], total bilirubin (TBIL), glucose and lactate; (4) Severity scores: Sequential Organ Failure Assessment (SOFA), Acute Physiology and Chronic Health Evaluation II (APACHE II), Simplified Acute Physiology Score II (SAPS II), and Charlson_index; (5) Coagulation and blood gas: International Normalized Ratio (INR), Prothrombin Time (PT), Partial Thromboplastin Time (PTT), Potential of Hydrogen (pH), Partial Pressure of Arterial Oxygen (PaO_2_), Partial Pressure of Arterial Carbon Dioxide (PaCO_2_); (6) Trauma severity scores: Injury Severity Score (ISS), Head Abbreviated Injury Scale (Head AIS), Chest Abbreviated Injury Scale (Chest AIS), Abdominal Abbreviated Injury Scale (Abdominal AIS), Shock Index (SI); (7) Trauma continuous: Red Blood Cell (RBC) transfusion_units, PaO_2_/FiO_2_ Ratio (PF), fibrinogen, d_dimer, Platelets Transfusion Units (PLT), Mechanical Ventilation Duration (MV), Glasgow Coma Scale (GCS) score, base_excess_trauma, and prehospital_time; (8) Trauma binary: Thoracic injury, Abdominal_injury, Pelvic_injury, Extremity injury; (9) Comorbidity information: diabetes mellitus (DM), hypertension (HTN), chronic kidney disease (CKD), liver disease, chronic obstructive pulmonary disease (COPD), and congestive heart failure (CHF); (10) Therapeutic interventions: mechanical ventilation (MV), vasoactive agents, continuous renal replacement therapy (CRRT), sedatives, antibiotics, fluid bolus administration, anticoagulation_use, and surgery within 24 h; (11) Hospitalization outcomes: length of ICU stay (LOS-ICU), total length of hospital stay (LOS-hospital). In terms of continuous variables, data were recorded including lactate, creatinine, respiratory rate, and other parameters representing the initial measurements obtained within 24 h following hospital admission. For binary variables, coding was performed as 0 or 1 according to the occurrence of the event.

### 2.4. Statistical Analysis

For variables with a missing rate below 30%, multiple imputation using regression models was adopted to generate 5 complete datasets, and one of them was randomly selected for analysis. Variables with a missing rate of ≥30% were removed from the study. Subsequently, patients were randomly divided into a training set (n = 589) and a validation set (n = 253) at a ratio of 7:3. In the training set, LASSO regression was applied to select important features. The regularization parameter was determined via 10-fold cross-validation with variables featuring non-zero coefficients retained. Subsequently, the Boruta algorithm was utilized to further screen variables, retaining features whose importance is significantly higher than that of random variables. Variables jointly selected by the two methods were used to construct the model, aiming to reduce overfitting and redundancy. To avoid multicollinearity, the Variance Inflation Factor (VIF) was calculated, and variables with a VIF > 5 were excluded [16]. A nomogram model was constructed using the filtered variables to predict 28-day all-cause mortality for patients with post-traumatic ARDS. The discriminative abilities of models including SOFA score, APACHE II score, XGBoost, Random forest, Decision tree and nomogram were evaluated by measuring the area under the receiver operating characteristic curve (AUROC). Calibration curves and the Hosmer–Lemeshow test were employed to evaluate the matching degree of “predicted risk” and “actual risk”. The decision curve analysis (DCA) curve was used to assess the net clinical benefit. The SHAP (SHapley Additive exPlanations) method was utilized to quantify the importance of each variable in this model, which provided a measure of the predictive contribution of each feature [17]. All statistical analyses were performed using R software (R 4.5.0). The data distribution was analyzed using the Shapiro–Wilk test. Continuous data were represented as mean ± standard deviation or median (interquartile range, IQR), and categorical variables were presented as frequencies and ratios (%). Non-parametric tests (Mann–Whitney U test or Kruskal–Wallis test) were employed for non-normally distributed or heteroscedastic data.

## 3. Results

### 3.1. Baseline Characteristics

We included 667 traumatic patients with ARDS, with 466 in the training cohort and 201 in the validation cohort. In the training set, 85.0% of patients survived, while 15.0% died. In the test cohort, 79.6% of patients survived, while 20.4% died. Non-survivors were older (69.10 ± 14.90 vs. 64.90 ± 12.95 years; *p* < 0.01), and had a higher Charlson index score (5.72 ± 2.08 vs. 4.74 ± 2.32; *p* < 0.01), Prehospital time (1.98 ± 1.12 h vs. 1.39 ± 0.88 h; *p* < 0.001), heart rate (100.08 ± 17.58 vs. 87.96 ± 16.42; *p* < 0.001), APACHE score (22.28 ± 8.59 vs. 18.81 ± 7.71; *p* < 0.001), SOFA score (11.72 ± 3.90 vs. 7.60 ± 3.58; *p* < 0.001), respiratory rate (24.39 ± 5.40 vs. 20.50 ± 5.22; *p* < 0.001), serum potassium (3.87 ± 0.46 mmol/L vs. 3.77 ± 0.49 mmol/L; *p =* 0.03), RBC units transfused (5.94 ± 3.96 units vs. 3.25 ± 2.21 units; *p* < 0.001), Shock index (0.94 ± 0.28 vs. 0.81 ± 0.22; *p* < 0.001), Lactate (3.52 ± 1.82 mmol/L vs. 2.62 ± 1.29 mmol/L; *p* < 0.001), Platelets transfusion units (1.94 ± 1.27 units vs. 0.54 ± 0.57 units; *p* < 0.001), Creatinine (2.59 ± 1.27 mg/dL vs. 1.41 ± 0.72 mg/dL; *p* < 0.001), D-dimer (4.54 ± 2.64 μg/mL vs. 2.77 ± 1.74 μg/mL; *p* < 0.001), Antibiotic use (1.00 [100%] vs. 0.95 [95%]; *p =* 0.03), Mechanical ventilation (0.81 [81%] vs. 0.67 [67%]; *p* < 0.001), Vasopressor use (0.73 [73%] vs. 0.54 [54%]; *p* < 0.001), and Renal replacement therapy use (0.24 [24%] vs. 0.15 [15%]; *p* = 0.03); they had lower temperature (37.19 ± 0.88 vs. 37.62 ± 0.82 °C; *p* < 0.001), Glasgow Coma Scale (7.03 ± 2.82 vs. 9.55 ± 3.09; *p* < 0.001), Albumin (2.53 ± 0.63 g/dL vs. 2.88 ± 0.61 g/dL; *p* < 0.001), and Base excess trauma (−5.17 ± 4.32 mmol/L vs. −2.32 ± 4.31 mmol/L; *p* < 0.001). Similar patterns were observed in the validation cohort (Table 1).

### 3.2. Variable Selection and Model Construction

**Figure 2:**
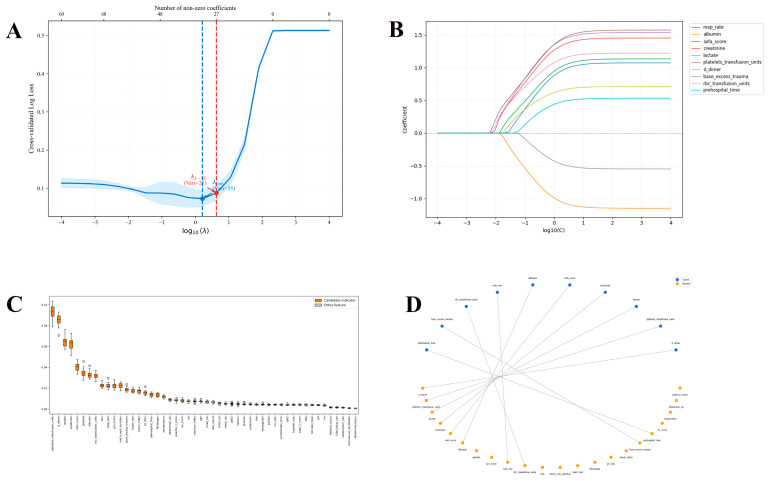
**The processing and selection of variables**. (**A**) Determination of the tuning hyperparameter (lambda) in LASSO regression through the application of the minimum criterion (dotted line on the left) and 1-SE criterion (dotted line on the right); (**B**) Coefficient distribution created from the log(lambda) sequence; (**C**) Predictor variable importance scores generated by the Boruta algorithm. The horizontal axis presents all predictor variables, with the vertical axis indicating the importance score as Z-scores; (**D**) The Boruta algorithm and LASSO regression were jointly employed to identify core variables, and the final selected features correspond to the intersection of the two approaches.

**Figure 3:**
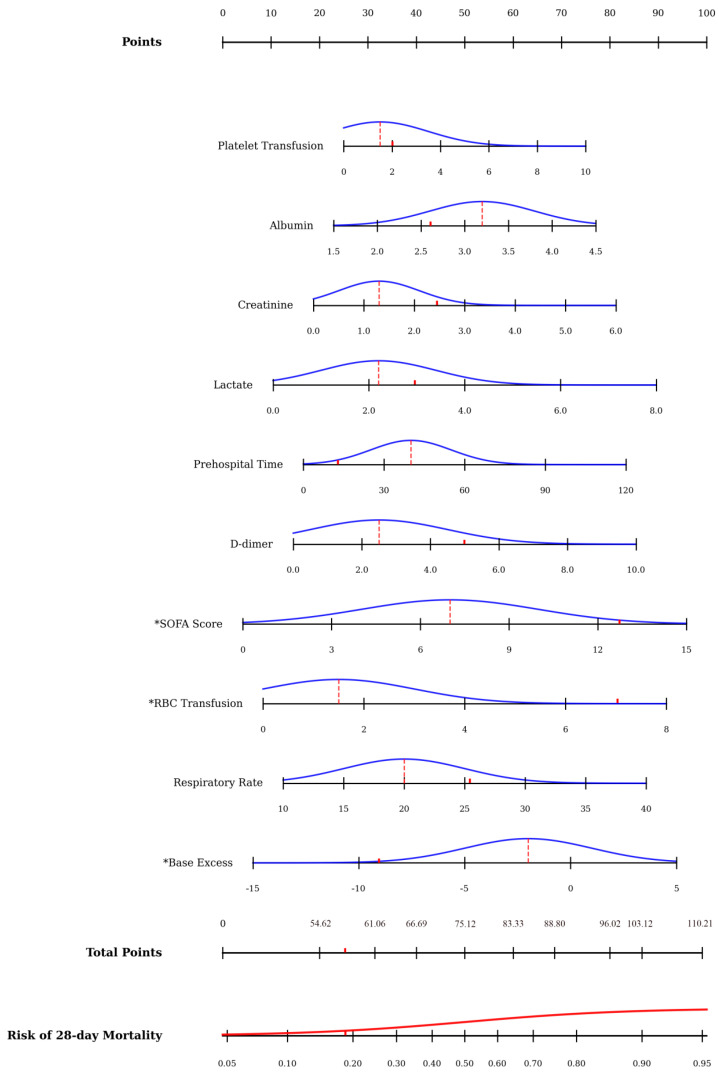
**Predicting the outcome by a nomogram**. A nomogram was developed based on the selected clinical parameters, then to quantify the probability of the outcome. A certain number of points was allocated to each variable, with the aggregated total score corresponding to the predicted probability displayed on the bottom scale. Asterisks denote the top three indicators; the red dashed line represents the measure of central tendency for the variable across the overall cohort; and the red solid line indicates the observed value for an individual patient.

In total, 10 predictive variables were identified from the initial set of clinical parameters by using LASSO regression and a Boruta algorithm. LASSO regression initially confirmed 27 parameters (Figure 2A,B), and the Boruta algorithm identified 22 important variables (Figure 2C). The selected features include prehospital time, base excess trauma, RBC transfusion units, Resp rate, albumin, SOFA Score, creatinine, lactate, platelets transfusion units, and D dimer (Figure 2D). Multicollinearity assessment suggested that the variance inflation factor values of all selected variables are less than 2 (Table 2). Based on these variables, a nomogram was constructed to predict 28-day all-cause mortality for traumatic patients with ARDS (Figure 3).

### 3.3. Predictive Model Performance

**Figure 4:**
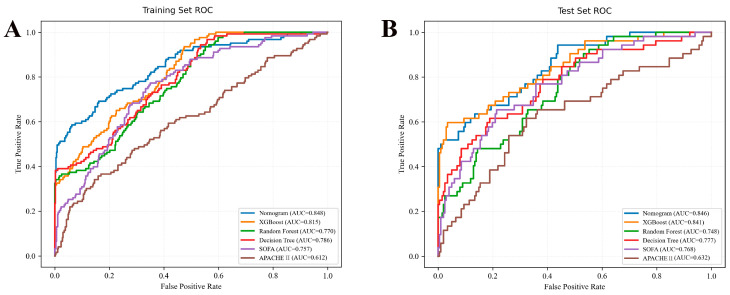
**ROC curves for predicting 28-day mortality in patients with post-traumatic ARDS**. (**A**) Training set and (**B**) Validation test comparing the performance of the Nomogram, SOFA, APACHE II, XGBoost, Random forest, and Decision tree. The Nomogram exhibited superior predictive ability in both cohorts.

The nomogram demonstrated superior discrimination (AUROC = 0.848) compared with SOFA (AUROC = 0.757), APACHE II (AUROC = 0.612), XGBoost (AUROC = 0.815), Random forest (AUROC = 0.770), and Decision tree (AUROC = 0.786) in the training set (Figure 4, *p* < 0.05). Similarly, the nomogram (AUROC = 0.846) had significantly better discrimination than SOFA (AUROC = 0.768), APACHE II (AUROC = 0.632), XGBoost (AUROC = 0.841), Random forest (AUROC = 0.748), and Decision tree (AUROC = 0.777) in the validation set (Figure 4, *p* < 0.05). Meanwhile, a sensitivity analysis of AUROC across different missing value imputation scenarios further verified the robustness of the nomogram’s discriminative ability, showing no significant AUROC fluctuations under various imputation strategies (Supplementary Figure S1).

### 3.4. Calibration and Interpretation of Predictive Model

**Figure 5:**
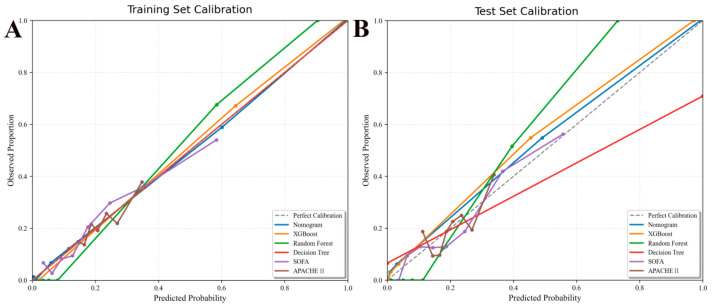
**Calibration curves for predicting 28-day mortality in patients with post-traumatic ARDS**. (**A**) Training set and (**B**) Validation test comparing the predicted probabilities of the Nomogram, SOFA, APACHE II, XGBoost, Random forest, and Decision tree against the observed probabilities. The dashed line represents the ideal calibration.

**Figure 6:**
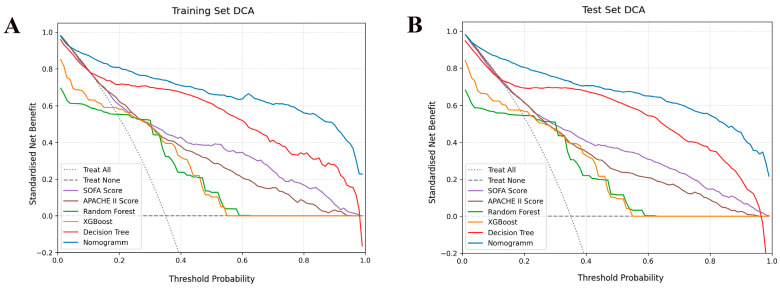
**Decision curve analysis (DCA) for predicting 28-day mortality in patients with post-traumatic ARDS**. (**A**) Training set and (**B**) Validation test comparing the net benefit of the Nomogram, SOFA, APACHE II, XGBoost, Random forest, and Decision tree.

**Figure 7:**
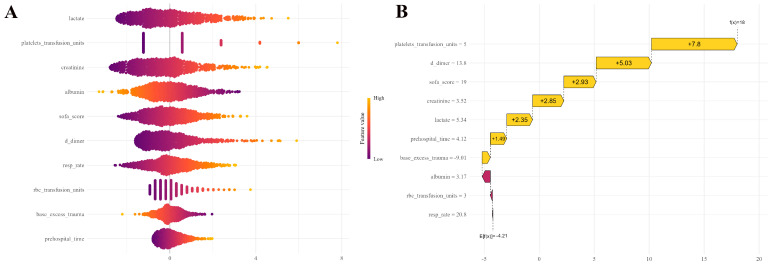
**SHAP analysis for predicting 28-day mortality in patients with post-traumatic ARDS**. (**A**) The distribution of SHAP values for per feature is depicted by the beeswarm plot; (**B**) The force plot illustrates the contributory role of individual features in a particular prediction.

The calibration curve is used to assess the level of consistency between the “predicted risk” and the “actual risk” within a predictive model. Calibration was evaluated through the Hosmer–Lemeshow test and calibration curves. The Hosmer–Lemeshow test showed good calibration in both the training set (χ^2^ = 3.63, df = 8, *p* = 0.889) and validation set (χ^2^ = 2.66, df = 8, *p* = 0.954), which suggests that there was no significant deviation between predicted and observed outcomes. The calibration curves demonstrated favorable consistency between actual and predicted probabilities across various predictive models (Figure 5). Decision curve analysis indicated that the net benefit of the Nomogram was higher than those of SOFA, APACHE II, XGBoost, Random forest, and Decision tree in a wide range of threshold probabilities (Figure 6). This indicates that Nomogram exhibits higher practicality in clinical decision-making and may offer superior decision support value to physicians. The SHAP beeswarm plot highlights that the core drivers were lactate, platelets transfusion units, creatinine, and D-dimer, which were the most influential predictors (Figure 7A). Conversely, albumin and base_excess_trauma reduced risk when their level were higher (Figure 7A). The SHAP force plot illustrates an individual patient’s prediction (Figure 7B).

## 4. Discussion

Post-traumatic ARDS is the most frequent manifestation following severe traumatic injury [18]. Post-traumatic ARDS is independently correlated to worse long-term quality-of-life outcomes and substantial mortality [19,20]. In patients with severe trauma, ARDS can occur in response to direct pulmonary insult such as pulmonary contusion, or indirect insults secondary to cellular injury and endothelial activation [21,22]. Systemic inflammatory storms induced by trauma, the disruption of the alveolar–capillary barrier, and cross-talk injury of multiple organ functions result in persistently high mortality in patients with post-traumatic ARDS [18,23]. Based on a large-sample clinical cohort, 10 key prognostic factors were identified through LASSO regression, Boruta algorithm, and VIF collinearity test, which covered trauma-specific scores, laboratory indicators, organ function status, and therapeutic interventions. The model achieves comprehensive coverage from “trauma severity-pathophysiological changes-clinical interventions-prognostic outcomes” and is simultaneously optimized for the disease-specific characteristics of ARDS, ensuring the reliability and reproducibility of the results. The results demonstrated that the model exhibited superior discriminative performance across both the training and validation cohorts, not only outperforming traditional scoring systems such as the SOFA score and APACHE II score, but also surpassing machine learning models including XGBoost and random forest. Calibration curves coupled with the Hosmer–Lemeshow test validated a close alignment between the model’s predicted risks and observed clinical outcomes, while DCA further confirmed that the model delivered consistent clinical net benefit across a broad spectrum of threshold probabilities. SHAP analysis further identified lactate, platelet transfusion units, creatinine, and D-dimer as the core risk factors driving mortality risk, while albumin and base excess in trauma emerged as independent protective factors. This study conducted a holistic assessment of model performance using multidimensional metrics, not only quantifying its discriminatory power and calibration efficacy but also resolving the “black box” conundrum of predictive models through SHAP analysis. Unlike prior trauma-related predictive models that suffer from the shortcoming of over-reliance on a single AUC value for evaluation, the nomogram developed in this study transforms complex regression models into an intuitive visual tool. Clinicians can swiftly compute patients’ mortality risk using routine laboratory indicators without the need for specialized algorithmic expertise, markedly enhancing the model’s clinical translation potential. Additionally, the trauma-specific parameters incorporated in this model (such as base excess trauma and RBC transfusion units) precisely align with the pathophysiological characteristics of post-traumatic ARDS. This enables earlier risk signal detection compared with conventional scoring systems. In summary, the nomogram effectively addresses the critical limitations of traditional tools, including poor early applicability and insufficient trauma-specificity, and thereby establishes a clinically actionable framework for early identification and targeted intervention.

This study enrolled adult ICU patients who underwent traumatic injury and had concurrent ARDS based on the MIMIC-IV database. The core predictors identified by SHAP analysis closely align with the unique pathophysiological progression of post-traumatic ARDS, and their predictive value can be fully elucidated through the underlying mechanisms. Lactate, as a sensitive biomarker for tissue hypoperfusion and metabolic disturbance [24], exerts a pivotal role in post-traumatic ARDS. Trauma-induced systemic inflammatory response leads to pulmonary microcirculatory dysfunction, alveolar epithelial injury, and vascular endothelial injury, thereby exacerbating tissue hypoxia, enhancing anaerobic metabolism and elevating substantial lactate production [25,26]. Multiple-organ impairment following trauma injury weakens the function of lactate clearance, forming a vicious cycle of “hypoxia-elevated lactate-exacerbated organ damage” [27,28]. The lactate level directly reflects the severity and poor prognosis, which is consistent with the fact that lactate serves a core role in the prognostic assessment of trauma patients. Traumatic injury is often accompanied by coagulation disorders and active hemorrhage [29]. Platelet transfusion serves as a pivotal intervention to maintain coagulation function and prevent life-threatening bleeding, whereas excessive or inappropriate transfusion may exacerbate lung injury through multiple pathways [30]. Activated platelets from massive transfusion can release inflammatory factors and chemokines, which further amplify the systemic inflammatory response, aggravate the disruption of the alveolar–capillary barrier, and exacerbate the pulmonary pathological changes in ARDS [30,31]. Meanwhile, the transfused platelets may be involved in microthrombosis formation, leading to the obstruction of pulmonary microcirculation, aggravating tissue hypoxia and metabolic disorders, thereby increasing the risk of death [32]. Systemic inflammatory response and tissue hypoperfusion induced by severe trauma directly impair renal function, which is reflected by elevated serum creatinine levels [30]. In turn, renal insufficiency can further exacerbate pulmonary pathological damage through inflammatory mediator accumulation and electrolyte disturbances [33]. Furthermore, elevated creatinine levels reflect the severity of organ function impairment following trauma, and persistent elevation indicates systemic metabolic disturbances and decline in repair capacity. D-Dimer derives from the plasmin-mediated degradation of cross-linked fibrin, which reflects the concomitant activation of both coagulation and fibrinolysis [34]. Elevated D-dimer indicates coagulation dysfunction following trauma [30]. Tissue injury induced by trauma activates the extrinsic coagulation pathway, promoting microthrombus formation. Microthrombi within the pulmonary microcirculation system further exacerbate alveolar ischemia and hypoxia, accelerating the progression of ARDS [35]. This is consistent with the pathological characteristic of mutual promotion between coagulation disorders and lung injury in post-traumatic complications. Albumin is a crucial protective factor against post-traumatic ARDS. Clinical studies have confirmed that trauma patients with hypoalbuminemia identified in the early post-traumatic phase exhibit a significantly increased risk of 28-day mortality [36]. Albumin can stabilize the structure of the vascular endothelial glycocalyx and maintain plasma colloid osmotic pressure, thereby reducing vascular leakage and tissue edema and alleviating pulmonary pathological damage [37]. Albumin can scavenge excessive reactive oxygen species (ROS) generated after trauma, bind and transport inflammatory mediators, regulate the intensity of the systemic inflammatory response, and thereby prevent sustained attacks on the lungs by the inflammatory storm. As the most abundant protein in plasma, albumin provides crucial support for lung protection in trauma patients through improving microcirculation, inhibiting pulmonary exudation, and regulating the inflammatory response. Base excess is closely associated with tissue perfusion and metabolic status. Normal base excess indicates milder tissue hypoxia and effectively controlled lactic acid metabolic disturbance after trauma. This can reduce acidosis-induced pulmonary vascular endothelial injury and vascular leakage, thereby mitigating pulmonary pathological damage [38]. Furthermore, stable base excess under dynamic monitoring can also indirectly reflect the body’s inflammation regulation capacity. Normal base excess can inhibit the release of inflammatory mediators, prevent the sustained disruption of the alveolar–capillary barrier by the inflammatory storm, and thereby further reduce the risk of ARDS progression [38].

Traditional assessment tools have inherent limitations in the population with post-traumatic ARDS, making it difficult to meet the demands of precise prognostic evaluation. The SOFA score focuses on the static assessment of organ dysfunction, but it fails to incorporate the cumulative effect of multi-site injuries induced by trauma and the associated pathophysiological specificity, thus being unable to fully capture disease heterogeneity. The APACHE II score is cumbersome to calculate, and its variable setup does not target the core pathological features of lung injury (e.g., alveolar–capillary barrier disruption, inflammation–coagulation crosstalk), leading to insufficient predictive specificity for this specific population. The nomogram constructed in this study effectively addresses the aforementioned limitations by integrating multi-dimensional variables, including trauma-related clinical indicators, laboratory parameters, organ function status, and therapeutic interventions. The AUC value of the nomogram is significantly higher than those of the SOFA score and APACHE II score, allowing for the more precise identification of high-risk patients and providing a more reliable basis for clinical decision-making. Compared with machine learning models such as XGBoost and random forests, the core advantages of the nomogram lie in clinical interpretability and practicality. Although machine learning models have a certain level of predictive performance, their black-box nature prevents clinicians from understanding the underlying logic of risk prediction, making it difficult for them to trust and apply these models in practical decision-making. In contrast, the nomogram translates complex algorithms into an intuitive scoring system. Combined with SHAP analysis, it can clarify the direction and magnitude of the risk contribution of individual variables to specific patients, thus having greater promotional value in the busy trauma care setting.

The nomogram constructed in this study can achieve the early risk stratification of patients with post-traumatic ARDS using easily accessible indicators within 24 h of admission, providing an intuitive and quantitative tool for clinical intervention decisions. From a pathophysiological perspective, this study supplements new operational indications and knowledge increments for the clinical management of post-traumatic ARDS and is applicable to multi-scenario clinical needs. Compared with the intervention logic of conventional ARDS treatments that only focus on “lung injury”, this model defines an individualized intervention indication based on the unique pathophysiological chain of “tissue hypoperfusion–inflammation activation–coagulation disorder” after trauma. Using the nomogram to guide intervention within this interval can avoid 20–40 unnecessary interventions per 100 patients, filling the gap of quantitative decision-making for traumatic scenarios in conventional treatment. The model is particularly valuable in special scenarios such as head and spinal cord trauma and damage control surgery. Patients with head and spinal cord trauma are prone to amplified lung injury due to neuroinflammation, while perioperative damage control surgery is associated with coagulation disorders and secondary injury risks. The trauma-specific indicators included in the nomogram (e.g., base excess trauma, platelet transfusion volume) can accurately capture pathophysiological abnormalities in such scenarios, providing a basis for early adjustment of intervention intensity (such as anticoagulant strategy and fluid management). Meanwhile, in the context of organ donation, the model can predict the risk of ARDS progression in advance to assist in optimizing donor evaluation and protection timing. In addition, this study deepens the understanding of the interaction between bleeding risk and ECMO hemodynamics. Patients with post-traumatic ARDS often face a triangular balance dilemma of “bleeding risk-anticoagulant demand-ECMO hemodynamic stability”. Indicators such as D-dimer and platelet transfusion volume included in the model can quantitatively reflect coagulation status and tissue perfusion level, providing quantitative references for adjusting anticoagulant intensity and early warning of bleeding risk during ECMO support. In summary, this study extends the model’s value from basic risk stratification to multi-scenario clinical decision-making, providing clinicians with a more comprehensive theoretical and practical basis for managing post-traumatic ARDS.

Despite the meaningful findings, there are unavoidable limitations that cannot be ignored. Firstly, the study adopted a retrospective design and relied on electronic health records from a clinical database. Some key clinical variables were not included in this analysis due to the limitations of database recording norms. The database is limited to document the binary status of mechanical ventilation without granular documentation of ventilatory strategies such as protective lung ventilation, prone positioning, PEEP levels, or tidal volumes, while these parameters represent critical determinants of pulmonary injury repair and clinical outcomes in post-trauma ARDS patients. Furthermore, the database only provides aggregate fluid administration volumes within the first 24 h of admission, while the PaO_2_/FiO_2_ ratio is restricted to a single baseline measurement. The absence of longitudinal monitoring data substantially limits the capacity to evaluate disease progression and therapeutic responsiveness in this population. The administration of specific pharmacologic interventions—including hemostatic/coagulation agents commonly administered in trauma settings such as tranexamic acid, fibrinogen concentrate, and recombinant activated factor VII (rFVIIa), as well as vasopressor dose titration—is limited in the database to binary documentation (“administered/not administered”). Granular quantitative data on dosage, the timing of administration, and sequential dose adjustments remain absent, thereby precluding the detailed analysis of dose–response relationships and temporal therapeutic windows critical for hemostatic resuscitation in trauma care. Secondly, the cohort data were derived from a single medical center, lacking external validation across hospitals of different levels and regions. However, regional disparities in trauma care quality may compromise the generalizability of the model. The model only incorporated static indicators within 24 h of admission, failing to consider dynamic monitoring data such as lactate clearance rate and daily changes in SOFA scores, which may underestimate the impact of disease progression on prognosis. Moreover, this study inevitably has endogeneity/intervention bias, which is also a core methodological limitation of such studies. Specifically, the clinical intervention measures for patients with post-traumatic ARDS (such as RBC/platelet transfusion dose, mechanical ventilation mode selection, vasopressor use intensity, etc.) are not randomly assigned but are jointly determined by the patient’s baseline disease severity and clinicians’ treatment decisions. This confounding association between intervention intensity and disease severity may lead to biases in the model’s evaluation of the association between intervention measures and prognosis. To mitigate this bias, this study has converted core intervention measures into quantitative or categorical variables for inclusion in the model, compressed the weight of confounding variables through the regularization mechanism of LASSO regression, controlled multicollinearity via VIF test, and conducted sensitivity analysis to verify model stability. However, these measures can only partially mitigate the bias and cannot completely eliminate the potential impact caused by the non-random assignment of interventions, which is also an important limitation of this study. In the future, this model could be further improved from multiple dimensions, such as conducting multicenter prospective studies to recruit patients with different trauma types from hospitals with varying levels of care quality to validate the model’s applicability in diverse populations and optimize the weights of predictors. Incorporating dynamic monitoring indicators to construct real-time updated dynamic prediction models would enhance the predictive accuracy for short-term outcomes. Combining multi-omics data with random features to explore in depth the molecular mechanisms by which core factors such as lactate and D-dimer influence the prognosis of post-traumatic ARDS would provide theoretical support for precision targeted therapy. Collectively, the nomogram constructed in this study integrates high predictive performance, strong interpretability, and favorable clinical practicality. It effectively addresses the shortcomings of traditional assessment tools and machine learning models, providing a reliable tool for the clinical early identification of high-risk patients and the formulation of individualized intervention strategies. The promotion and application of this model are expected to optimize the clinical management processes of post-traumatic ARDS, reduce patient mortality, and improve long-term prognosis.

## Figures and Tables

**Table 1:** Baseline characteristics and comparison of training and validation cohorts.

Characteristic	Training Set (n = 466)		*p* Value (Train)	Test Set (n = 201)		*p* Value (Test)	*p* Value (Overall)
	Survival (n = 396)	Non-Survival (n = 70)		Survival (n = 160)	Non-Survival (n = 41)		
**Demographic/baseline characteristic**							
Age	64.90 ± 12.95	69.10 ± 14.90	*p* < 0.001	63.95 ± 14.62	70.11 ± 12.10	*p* < 0.001	*p* > 0.05
Male proportion	0.61 (61%)	0.60 (60%)	0.92	0.53 (53%)	0.58 (58%)	0.68	*p* > 0.05
Charlson index	4.74 ± 2.32	5.72 ± 2.08	*p* < 0.001	4.73 ± 2.26	5.99 ± 2.28	*p* < 0.001	*p* > 0.05
Prehospital time	1.39 ± 0.88	1.98 ± 1.12	*p* < 0.001	1.29 ± 0.79	1.69 ± 1.01	*p* < 0.01	*p* > 0.05
**Disease severity**							
APACHE score	18.81 ± 7.71	22.28 ± 8.59	*p* < 0.001	18.58 ± 7.65	22.28 ± 8.68	0.01	*p* > 0.05
SOFA score	7.60 ± 3.58	11.72 ± 3.90	*p* < 0.001	7.79 ± 3.47	11.78 ± 3.78	*p* < 0.001	*p* > 0.05
**Vital sign**							
Heart rate	87.96 ± 16.42	100.08 ± 17.58	*p* < 0.001	89.77 ± 14.86	98.57 ± 16.99	0.00	*p* > 0.05
Systolic blood pressure	111.41 ± 16.78	113.44 ± 17.13	0.24	111.64 ± 17.50	111.96 ± 17.00	0.90	*p* > 0.05
Mean arterial pressure	77.77 ± 10.35	77.14 ± 11.25	0.57	77.47 ± 11.21	75.98 ± 11.03	0.39	*p* > 0.05
Shock index	0.81 ± 0.22	0.94 ± 0.28	*p* < 0.001	0.82 ± 0.22	1.04 ± 0.33	*p* < 0.001	*p* > 0.05
Respiratory rate	20.50 ± 5.22	24.39 ± 5.40	*p* < 0.001	19.68 ± 4.75	23.83 ± 5.50	*p* < 0.001	*p* > 0.05
Temperature	37.62 ± 0.82	37.19 ± 0.88	*p* < 0.001	37.57 ± 0.80	37.36 ± 0.89	0.12	*p* > 0.05
Glasgow Coma Scale	9.55 ± 3.09	7.03 ± 2.82	*p* < 0.001	9.26 ± 2.97	6.97 ± 2.75	*p* < 0.001	*p* > 0.05
**Laboratory parameter**							
White blood cell count	13.15 ± 5.22	14.24 ± 5.69	0.06	13.62 ± 5.50	13.40 ± 5.76	0.81	*p* > 0.05
Blood glucose	155.42 ± 53.11	162.06 ± 51.43	0.21	155.04 ± 56.78	168.31 ± 50.42	0.10	*p* > 0.05
Serum sodium	136.53 ± 5.53	135.55 ± 5.49	0.08	137.13 ± 5.34	137.10 ± 6.01	0.98	*p* > 0.05
Serum potassium	3.77 ± 0.49	3.87 ± 0.46	0.03	3.86 ± 0.49	3.87 ± 0.50	0.92	*p* > 0.05
Lactate	2.62 ± 1.29	3.52 ± 1.82	*p* < 0.001	2.60 ± 1.44	4.24 ± 1.96	*p* < 0.001	*p* > 0.05
Creatinine	1.41 ± 0.72	2.59 ± 1.27	*p* < 0.001	1.42 ± 0.78	2.86 ± 1.37	*p* < 0.001	*p* > 0.05
Albumin	2.88 ± 0.61	2.53 ± 0.63	*p* < 0.001	2.88 ± 0.63	2.48 ± 0.64	*p* < 0.001	*p* > 0.05
D-dimer	2.77 ± 1.74	4.54 ± 2.64	*p* < 0.001	2.76 ± 1.93	4.96 ± 2.70	*p* < 0.001	*p* > 0.05
Base excess trauma	−2.32 ± 4.31	−5.17 ± 4.32	*p* < 0.001	−2.55 ± 4.59	−5.78 ± 4.57	*p* < 0.001	*p* > 0.05
**Therapeutic intervention**							
RBC units transfused	3.25 ± 2.21	5.94 ± 3.96	*p* < 0.001	3.00 ± 2.07	6.46 ± 3.96	*p* < 0.001	*p* > 0.05
Platelets transfusion units	0.54 ± 0.57	1.94 ± 1.27	*p* < 0.001	0.49 ± 0.58	1.91 ± 1.23	*p* < 0.001	*p* > 0.05
Renal replacement therapy use	0.15 (15%)	0.24 (24%)	0.03	0.14 (14%)	0.27 (27%)	0.04	*p* > 0.05
Mechanical ventilation	0.67 (67%)	0.81 (81%)	*p* < 0.001	0.66 (66%)	0.88 (88%)	0.00	*p* > 0.05
Sedation use	0.38 (38%)	0.42 (42%)	0.42	0.46 (46%)	0.52 (52%)	0.57	*p* > 0.05
Antibiotic use	0.95 (95%)	1.00 (100%)	0.03	0.94 (94%)	1.00 (100%)	0.15	*p* > 0.05
Heparin use	0.19 (19%)	0.25 (25%)	0.19	0.20 (20%)	0.21 (21%)	0.99	*p* > 0.05

**Table 2:** Variance inflation factor between variables.

Feature	VIF
lactate	1.172307216
platelets_transfusion_units	1.241154634
creatinine	1.204295785
albumin	1.098638376
sofa_score	1.146582326
d_dimer	1.23720193
resp_rate	1.059255411
rbc_transfusion_units	1.119858414
base_excess_trauma	1.052197568
prehospital_time	1.067971275

## Data Availability

The data can be acquired from the Medical Information Mart for Intensive Care IV (MIMIC-IV) version 3.0 database (https://physionet.org/content/mimiciv/3.0/ (accessed on 26 October 2025)).

## Supplementary Materials

The following supporting information can be downloaded at: https://www.mdpi.com/article/10.3390/jcm15052073/s1, Figure S1: Sensitivity Analysis for AUROC across Imputation Scenarios.
