# Supplementary material for: Development and Validation of a Multidimensional Predictive Model for 28-Day Mortality in Patients with Post-Traumatic Acute Respiratory Distress Syndrome
Source: J Clin Med. 2026 Mar 9;15(5):2073. doi: 10.3390/jcm15052073 (PMC12986380; doi:10.3390/jcm15052073)
Supplement: Supplementary file 1 [file jcm-15-02073-s001.zip › jcm-4080490-supplementary.pdf]

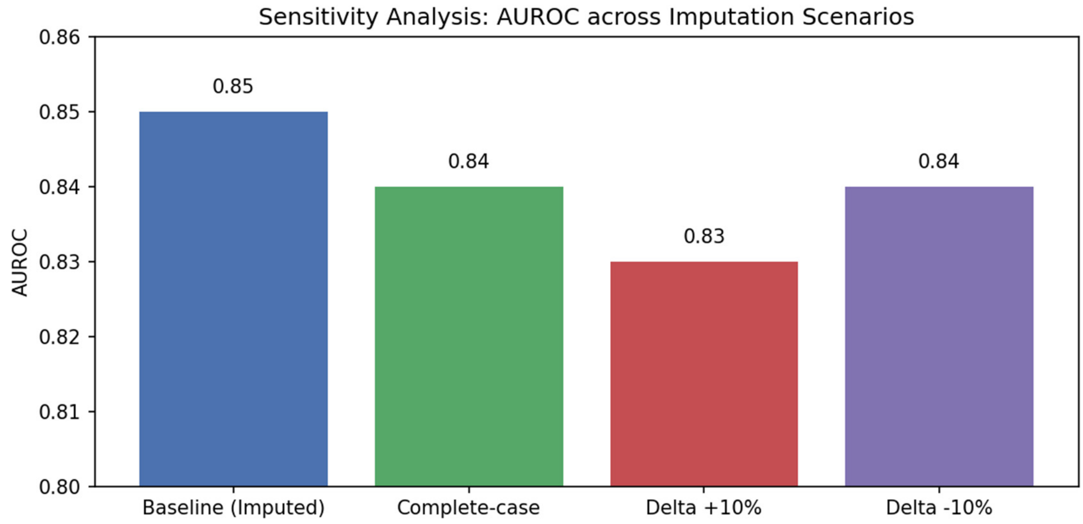

Supplementary Figure S1. Sensitivity Analysis for AUROC across Imputation Scenarios

AUROC values of the predictive model under different imputation and data scenarios.

The model shows stable discriminative performance across all scenarios.
